# The effects of fermented *Astragalus* polysaccharides on the growth performance, antioxidant capacity and intestinal health of broilers

**DOI:** 10.3389/fvets.2025.1530117

**Published:** 2025-02-25

**Authors:** Zhenkun Liu, Huaidan Zhang, Xianxin Chen, Weiwei Yu, Shiyi Li, Lijuan Kang, Songlin Li, Yilong Jiang, Xinhong Zhou

**Affiliations:** ^1^Chongqing Three Gorges Vocational College, Chongqing, China; ^2^Leshan Academy of Agriculture Science, Leshan, China; ^3^Institute of Animal Nutrition, Sichuan Agricultural University, Chengdu, China; ^4^School of Life Science and Engineering, Southwest University of Science and Technology, Mianyang, China

**Keywords:** fermented *Astragalus* polysaccharides, antioxidant, intestinal barrier, intestinal microbiota, broilers

## Abstract

This study aimed to investigate the effects of fermented *Astragalus* polysaccharides(FAP) on the growth performance, antioxidant capacity and intestinal health of broilers. A total of 1,080 Cyan-shank Partridge chickens were divided into 4 groups, with 6 replicates per group and 45 chickens per replicate. Add 0% (T1), 0.2% (T5), 0.4% (T6) and 0.6% (T7) of FAP to the basal diet, respectively. The trial lasted for 42 days. The results indicated that, compared to the T1 group, FW and ADG of broilers in each treatment group were significantly increased (*p < 0.05*). The slaughter rates of the T6 and T7 groups were significantly higher compared to the T1 group, meanwhile, the carcass yields of the T5, T6, and T7 groups were notably enhanced (*p < 0.05*). Compared with T1 group, the activities of CAT, GSH-Px and the content of T-AOC in T6 and T7 groups were increased (*p* < 0.05), while the content of MDA was decreased (*p* < 0.05). All groups exhibited significantly VH and VH/CD in the duodenum compared to the T1 group (*p < 0.05*). Compared with the T1 group, the relative mRNA expression levels of *ZO-1* and *Claudin* in the jejunal mucosa of broilers in all groups were significantly up-regulated, while the expressions of *IL-1β*, *IL-6*, *TNF-α*, and *IFN-γ* were down-regulated (*p < 0.05*). 16S rDNA sequencing analysis revealed that at the phylum level, the abundance of Verrucomicrobiota in the T6 group was significantly increased compared to the T1 group (*p < 0.05*). Cyanobacteria, Nitrospirota, Elusimicrobiota, and Acidobacteriota were unique to the T6 group, while Cyanobacteria and Elusimicrobiota were unique to the T5 group compared to the T1 group. At the genus level, the abundance of *Desulfovibrio* was significantly reduced in the T6 group compared to the T1 group (*p < 0.05*). Additionally, fermented *Astragalus* polysaccharides increased the abundance of *Bacteroidota, Campilobacterota*, *Deferribacterota*, *Firmicutes*, *Fusobacteriota*, *Proteobacteria*, and *Spirochaetota* (*p* < 0.05). The LEfSe analysis found that *Clostridia_vadinBB60_group* and *Comamonas* were identified as potential biomarkers. Overall, feeding fermented *Astragalus* polysaccharides can enhance the growth performance, slaughter characteristics, and antioxidant capacity of broiler chickens by modulating the gut microbiota and strengthening intestinal barrier function.

## 1 Introduction

*Astragalus* is a perennial herbaceous legume widely distributed in temperate regions (1). Astragalus polysaccharide (APS) is one of the main active components of *Astragalus* and has been widely used in animals due to its solubility characteristics (2). Studies have shown that APS not only promotes the colonization of beneficial bacteria in the intestinal tract, but also effectively inhibits the growth of harmful pathogens, thus maintaining intestinal health. In addition, APS has the ability to scavenge Reactive Oxygen Species (ROS) and reduce intestinal inflammation, which further improves intestinal barrier function (3). Also, APS can promote the secretion of immunoglobulin A (IgA), which enhances mucosal immunity (4, 5). APS can activate lymphocyte receptor signaling pathway, which enhances the body’s adaptive and innate immunity, thus exerting its antimicrobial effects (6). APS if regulates gene expression of *VCAM1, RELA, CDK2* and other genes to treat pulmonary fibrosis effects (7). In addition, incorporation of APS into the diet promotes growth, improves feed conversion efficiency, and enhances the antioxidant capacity of the organism, e.g., by increasing *superoxide dismutase* (SOD) and *catalase* (CAT) activities (8, 9). In mammalian aquaculture, the addition of APS has been shown to inhibit the lipopolysaccharide-induced *mitogen-activated protein kinase* (*MAPK*) and nuclear *factor-κB* (NF-κB) inflammatory pathways, reduce the expression of intestinal inflammatory factors, improve the morphology of intestinal villi, and reduce intestinal damage, thereby enhancing intestinal immunity and productivity (10). Herbal polysaccharides play a major role in metabolic diseases and have wide applicability to livestock health. Therefore, the therapeutic potential of herbs is enormous (11).

A wide variety of strains are available for the fermentation of traditional Chinese medicine, and we evaluated them in terms of the convenience of testing conditions and the broad spectrum of antimicrobial substances. The results showed that *Bacillus licheniformis* had excellent antimicrobial capacity and tolerance compared to other probiotics and was able to maintain activity at room temperature. It was also shown that the products fermented by *Bacillus licheniformis* may be novel candidate food additives with impact on the farming industry (12). Therefore, we chose *Bacillus licheniformis* as herbal fermentation agent. *Bacillus licheniformis* is widely used in farming (13) and has been shown to possess anti-inflammatory properties (14). Studies on porcine intestinal epithelial cells have shown that *B. licheniformis* can improve the barrier function of intestinal epithelial cells, regulate the expression of *interleukin 6* (*IL-6*) and *interleukin 8* (*IL-8*), and reduce intracellular Reactive Oxygen Species (ROS) production, resulting in enhanced intestinal function and conversion efficiency (15). In addition, it increases the abundance of *B. mitoticus* in the feces of weaned piglets, thereby stabilizing the intestinal microbiota (16). In poultry farming, the addition of *Bacillus licheniformis* to feed has been found to enhance growth performance, immune status, and antioxidant capacity, increase short-chain fatty acid production, and modulate the intestinal microbiota in *broilers* (17). Previous studies have found that fermented *astragalus* polysaccharides inhibit the expression of the pro-inflammatory cytokines tumor necrosis factor *α* (*TNF-α*) and *interleukin 6* (*IL-6*), thereby attenuating tissue inflammatory injury (18).

However, within the context of restricted use of antibiotics, the exploration for safe and effective antibiotic substitutes has emerged as a central focus in the livestock and poultry breeding industry. Fermented polysaccharides, as a novel green feed additive, possess broad application prospects. Current research on the application of astragalus polysaccharides has revealed that astragalus polysaccharides can pass through the Reactive Oxygen Species (ROS) pathway in chicken embryonic fibroblasts thereby impeding cadmium-induced autophagic damage and protect chicken peripheral blood lymphocytes through the *MDA5/NF-κB* pathway.In this study (19, 20). We hope to provide a theoretical basis for elucidating the mechanism of action of *Bacillus licheniformis* fermented Astragalus polysaccharide in poultry farming by combining *Bacillus licheniformis* with APS for the first time for fermentation treatment and further exploring its effects on growth performance, antioxidant capacity and intestinal health of broilers based on the results of the previous work.

## 2 Materials and methods

### 2.1 Experimental feed

*Bacillus licheniformis* was inoculated into LB liquid medium and incubated at 37°C for 24 h to obtain activated bacterial liquid. Then, APS (APS: bacterial liquid = 7:3) was added to *Bacillus licheniformis* (108 CFU/mL) and incubated at 37°C for 24 h. The FAP was then lyophilized and stored in well-sealed glass ampoules protected from moisture by silica gel desiccant. The dried FAP was mixed thoroughly with different ratios (0.2, 0.4, 0.6%) of the basal diet (Table 1), and the temperature and pH conditions were strictly controlled throughout the experimental cycle to avoid any imbalance of FAP.

**Table 1:** Formulation and nutrient composition of basal diets.

Ingredient	Base diet	Nutritional levels^b^	
Corn	60.00	Metabolic energy (MJ/kg)	12.15
Soybean meal	20.20	Crude protein	18.80
Wheat bran	5.00	Calcium	0.95
Shell powder	8.00	Phosphorus	0.65
Ca(HCO_3_)_2_	2.00	Ca	0.73
Lysine	0.10	P	0.37
Methionine	0.17	Lysine	0.99
NaCl	0.33	Methionine	0.44
Soybean oil	1.20		
Premix^a^	3.00		
Total	100		

a The premix provides the following nutrients per kilogram of feed: biotin (0.1 mg), pantothenic acid (20 mg), folic acid (1.5 mg), niacin (50 mg), zinc (110 mg), iodine (0.6 mg), copper (9 mg), iron (100 mg), selenium (0.16 mg), manganese (100 mg), vitamin A (15,000 IU), vitamin D3 (2,500 IU), vitamin E (20 mg), vitamin K3 (3 mg), vitamin B1 (3 mg), vitamin B2 (8 mg), vitamin B6 (7 mg), and vitamin B12 (0.03 mg).

b All values were measured except for metabolizable energy.

### 2.2 Experiment design

**Figure 1:**
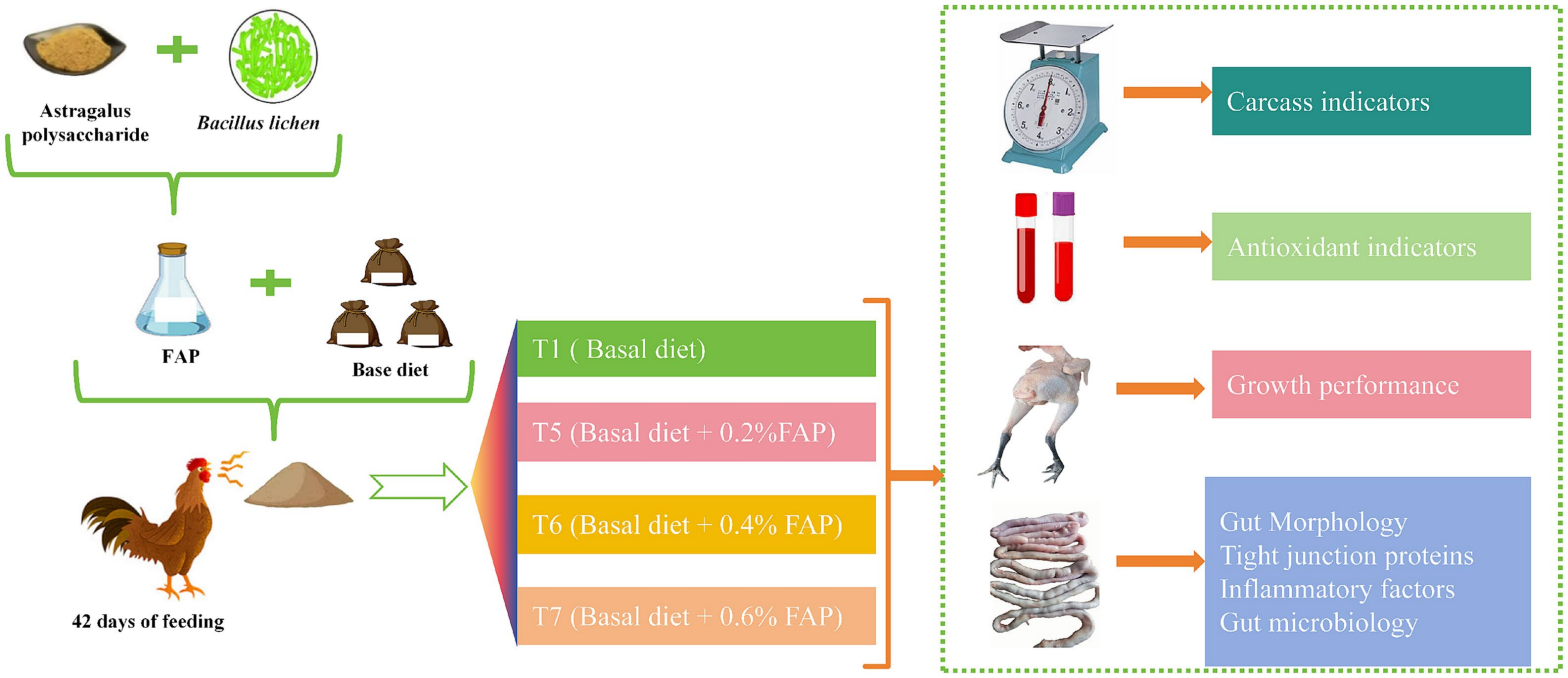
Experimental mechanism.

A total of 1,080 healthy male Cyan-shank Partridge chickens，free of vertically transmitted diseases and in uniform body condition, weighing 3,135–3,335 g, were selected for this study. Divided into four groups, with 6 replicates per group and 45 chickens per replicate. Each replicate was housed in a cage measuring 220 cm × 200 cm × 100 cm. The T1 group received a basal diet, while the T5, T6, and T7 groups received basal diets supplemented with 0.2, 0.4, and 0.6% FAP, respectively. The experimental period lasted for 42 days. The chickens were fed twice daily and had access to natural light, water, and feed throughout the study. The temperature inside the chicken house was maintained between 18°C and 25°C. The experimental design of this study is shown in Figure 1.

### 2.3 Sample collection

At the end of the experiment, one chicken was randomly selected from each replicate, and 5 mL of blood was collected from the wing vein. Serum was separated by centrifugation for serum antioxidant index analysis. The chickens were then euthanized using carbon dioxide and dissected to measure carcass traits. Samples (approximately 2 cm each) were taken from the middle sections of the duodenum, jejunum, and ileum, rinsed with physiological saline to remove contents, and preserved in 4% paraformaldehyde for intestinal morphology analysis. Jejunal mucosa and cecal content samples were collected, rapidly frozen in liquid nitrogen, and stored at −80°C for further analysis.

### 2.4 Measurement and methods

#### 2.4.1 Growth performance

Daily feed intake and leftover feed were recorded throughout the experimental period. On days 1 and 42 of the trial, chickens were weighed after a 12-h fasting period to determine initial and final weights. Based on feed consumption, the following parameters were calculated:

Average Daily Feed Intake (ADFI) (g) = Total feed consumption / Number of trial days.
Average Daily Gain (ADG) (g) = (Final weight - Initial weight) / Number of trial days.
Feed Conversion Ratio (F/G) = Total feed consumption / (Final weight - Initial weight).

#### 2.4.2 Carcass quality

At the end of the experiment, one chicken was randomly selected from each replicate, euthanized using carbon dioxide, and quickly dissected to assess slaughter performance. The measured parameters were as follows:

Slaughter Yield (%) = 100% × carcass weight / live weight.
Eviscerated Yield (%) = 100% × eviscerated weight / live weight.
Dressed Yield (%) = 100% × dressed weight / live weight.
Leg Muscle Yield (%) = 100% × leg muscle weight / dressed weight.
Breast Muscle Yield (%) = 100% × breast muscle weight / dressed weight.

#### 2.4.3 Serum antioxidant capacity

Following the kit instructions (Jiancheng Biotech Ltd., Nanjing, China), Serum catalase (CAT), glutathione peroxidase (GSH-Px), superoxide dismutase (SOD), total antioxidant capacity (T-AOC), and malondialdehyde (MDA) activities or levels were determined using a Multiskan GO microplate spectrophotometer (Multiskan GO Microplate Spectrophotometer without cuvette, Thermo Fisher Scientific, Japan).

#### 2.4.4 Intestinal morphology

After immersion in 4% paraformaldehyde, the duodenum, jejunum and ileum experienced dehydration, transparency, embedding, and sectioning into 5-micrometer slices. Subsequently, the sections were dewaxed and rehydrated. Hematoxylin and eosin (HE) staining was performed followed by dehydration, clearing, and mounting. Images were captured using an optical microscope. Using Image Pro Plus 6.0 (Media Cybernetics, Inc. Silver Spring, Maryland, USA) software, villus height (VH) and crypt depth (CD) were measured to assess intestinal morphology.

#### 2.4.5 Prediction of action site of Astragalus polysaccharide

The structural formula of astragalus polysaccharide was drawn by Chem Draw. The structure of *Occludin* (*1WPA, Homo sapiens*) and *ZO-1 protein* (*3LH5, Homo sapiens*) were retrieved from PDB database.^1^ Molecular docking was performed using Autodock Vina software, and Discovery Studio software was utilized to optimize the docking results output (21).

#### 2.4.6 Gene expression analysis of jejunal mucosa

Jejunal mucosa samples were ground in liquid nitrogen and total RNA was extracted from 50 to 100 mg of tissue according to the manufacturer’s instructions TaKaRa MiniBEST Universal RNA Extraction Kit (N0.9769). RNA integrity and concentration were assessed using 1% agarose gel electrophoresis and a micro UV spectrophotometer (Thermo Fisher Scientific, Japan) to ensure that the *OD260/OD280* ratio was between 1.8 and 2.1. cDNA was synthesized from RNA samples according to the manufacturer’s protocol (RR047A, TaKaRa PrimeScript™ RT reagent Kit with gDNA Eraser, TaKaRa). cDNA was synthesized using the CFX96 Real-Time PCR (Bio-Rad Laboratories, Inc., Hercules, California, USA). Real-time quantitative PCR amplification was performed using the *CFX96* Real-Time PCR (Bio-Rad Laboratories, Inc., Hercules, California, USA) detection system. The primers are shown in Table 2, and *GAPDH* was used as the reference gene.

**Table 2:** Primer sequences.

Genes	Sequence 5′ − 3′	GenBank number
*GAPDH*	F: GGAAAGTCATCCCTGAGCTGAAT	NM_204305.1
	R: GGCAGGTCAGGTCAACAACA	
*ZO-1*	F: AATACCTGACTGTCTTGCAG	XM_015278975.1
	R: TAAAGAAGGCTTTCCCTGAC	
*IFN-γ*	F: AAAGCCGCACATCAAACACA	NM_205149.1
	R: GCCATCAGGAAGGTTGTTTTTC	
*Occludin*	F: GCAGATGTCCAGCGGTTACTAC	NM_205128.1
	R: CGAAGAAGCAGATGAGGCAGAG	
*Claudin*	F: CATACTCCTGGGTCTGGTTGGT	NM_001013611.2
	R: GACAGCCATCCGCATCTTCT	
*IL-1β*	F: ACTGGGCATCAAGGGCTA	XM_015297469
	R: GGTAGAAGATGAAGCGGGTC	
*IL-6*	F: GAAATCCCTCCTCGCCAATCT	XM_0152812832
	R: CCTCACGGTCTTCTCCATAAACG	
*TNF-α*	F: TGTGTATGTGCAGCAACCCGTAGT	NM_204267
	R: GGCATTGCAATTTGGACAGAAGT	

#### 2.4.7 Sequencing of cecal contents 16S rDNA

Microbial DNA was extracted from 200 mg of cecal samples from each group using the MagPure Soil DNA LQ kit (Guangdong Magen, China), following the manufacturer’s instructions. The V3-V4 hypervariable region of the bacterial 16S rRNA gene was amplified using TksGflflex DNA Polymerase (Takara, R060B) and universal primers 343F (5’-TACGGRAGGCAGCAG-3′) and 798R (5’-AGGGTATCTAATCCT-3′) in a 30 μL reaction mixture. Sequencing was performed on the Illumina NovaSeq6000 platform with paired-end reads of 250 bases per cycle. The sequencing of the 16S rDNA gene amplicon fragments and subsequent bioinformatics analysis were conducted by Shanghai OE Biotech Co., Ltd.

#### 2.4.8 Data analysis

Alpha and beta diversity analyses were conducted using QIIME software. The alpha diversity of the samples was assessed using indices including Chao1 and Shannon. Unweighted UniFrac distance matrices calculated in R were used for unweighted UniFrac principal coordinates analysis (PCoA) to evaluate the beta diversity of the samples. Differential analysis was performed using ANOVA/Kruskal Wallis/T test/Wilcoxon statistical methods based on R packages. LEfSe was used for differential analysis of species abundance profiles. The relative expression of target gene mRNA was calculated using the 2^-ΔΔCt^ method. All data were initially processed using Excel and subsequently analyzed using SPSS 23.0. One-way ANOVA was performed to analyze the differences among groups, followed by Duncan’s multiple range test for post-hoc comparisons. Differences were considered statistically significant at *p < 0.05*. Results are presented as mean values ± standard error.

## 3 Results

### 3.1 Effects of FAP on growth performance of chickens

Table 3 illustrates the impact of the FAP on the growth performance of the chickens. Compared to the T1 group, the addition of FAP significantly enhanced the FW and ADG, while notably reducing the F/G (*p* < 0.05). Furthermore, in comparison to the T1 group, the ADFI in the T7 group was significantly decreased (*p* < 0.05).

**Table 3:** Effects of FAP on growth performance of chickens.

Items	T1	T5	T6	T7	SEM	*P*-value
IW (kg)	1.48	1.47	1.51	1.47	0.02	0.073
FW (kg)	2.92^b^	3.14^a^	3.23^a^	3.19^a^	0.02	*<0.01*
ADG(g)	34.40^b^	39.67^a^	40.77^a^	40.98^a^	0.87	0.013
ADFI(g)	156.70^a^	152.13^ab^	148.98^ab^	146.99^b^	0.95	*<0.01*
F/G	4.60^a^	3.87^b^	3.67^b^	3.61^b^	0.55	*<0.01*

Means with different lowercase are significantly different (*P* < 0.05, Duncan’s multiple comparisons).

### 3.2 Effects of FAP on slaughter performance of chickens

**Figure 2:**
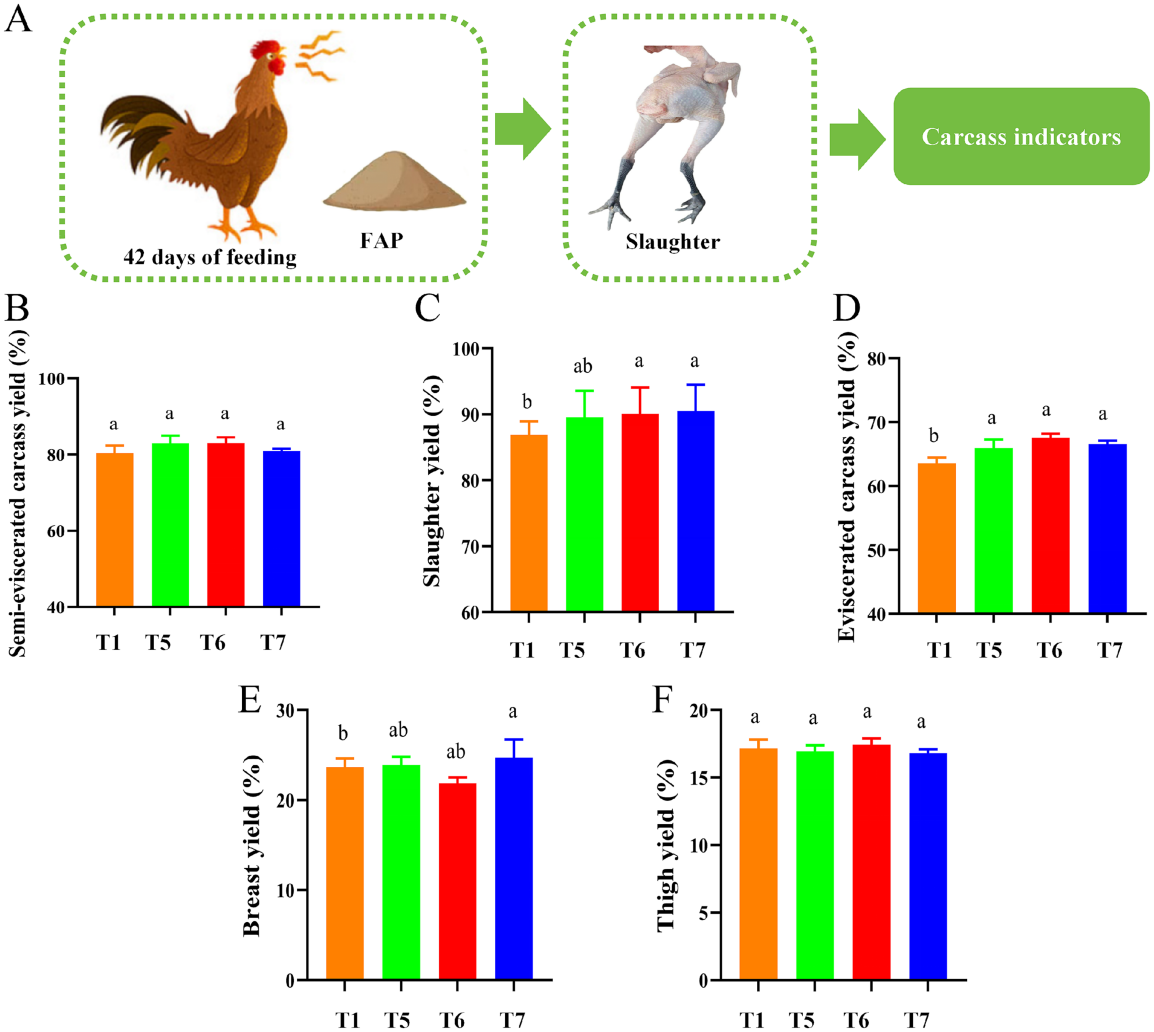
Assessment of chicken slaughter performance. **(A)** Diagram of slaughter performance assessment mechanism. **(B)** Semi-skinless carcass yield. **(C)** Slaughter yield. **(D)** Exsanguinated carcass yield. **(E)** Breast yield. **(F)** Thigh yield. Different lowercase letters indicate significant differences (*p < 0.05,* Duncan multiple comparisons).

**Figure 3:**
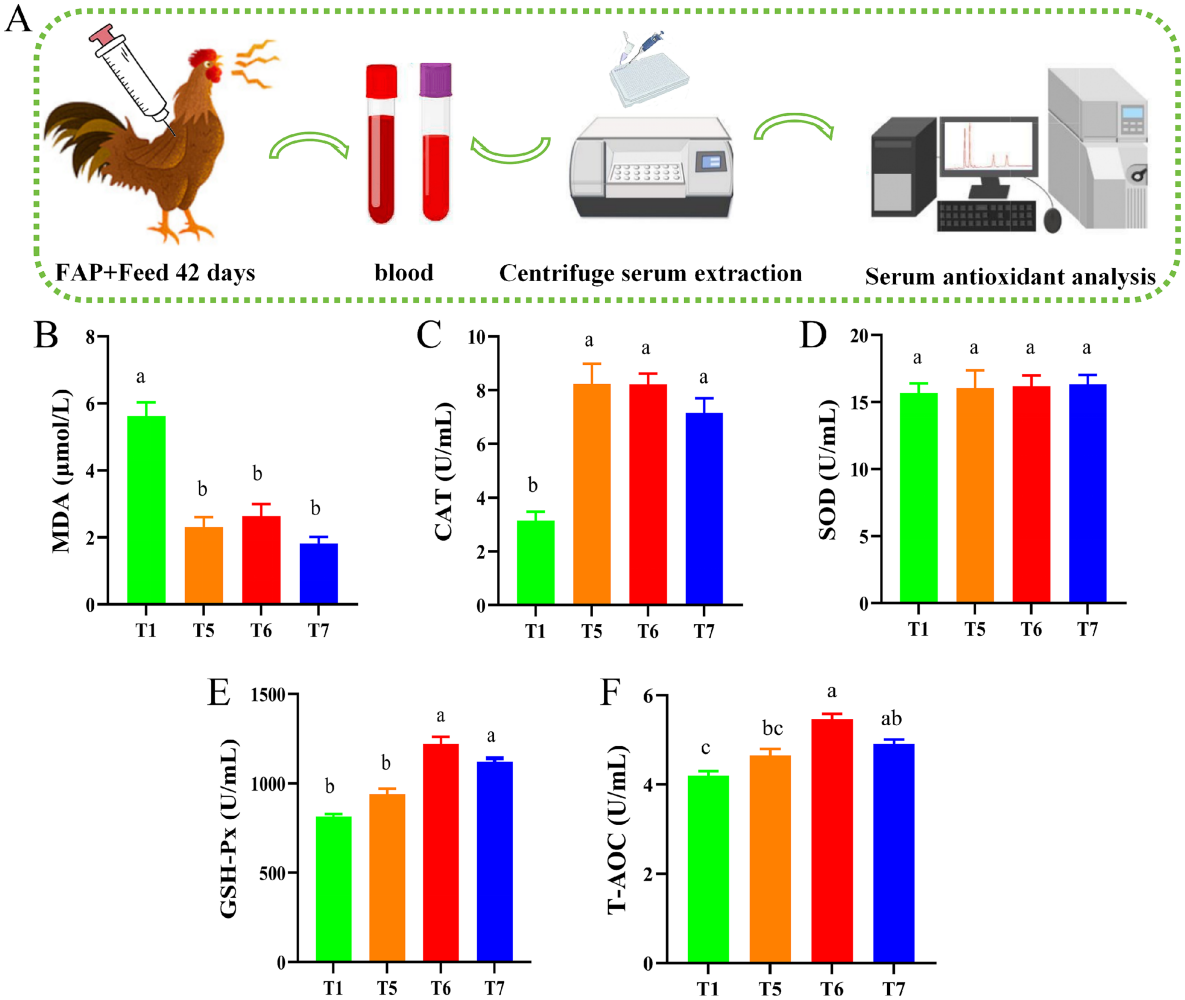
Chicken serum antioxidant capacity assays. **(A)** Diagram of serum biochemical assay mechanism. **(B)** MDA content. **(C)** CAT activity. **(D)** SOD activity. **(E)** GSH-Px activity. **(F)** T-AOC level. Different lowercase letters indicate significant differences (*p* < 0.05, Duncan multiple comparisons).

Figure 2A shows the slaughter experiment process. According to the results presented in Figure 3, FAP had a significant impact on the slaughter performance of the chickens. Compared to the T1 group, the slaughter weight in the T6 and T7 groups was significantly increased (*p* < 0.05). Additionally, the visceral carcass yield in the T5, T6, and T7 groups showed a remarkable increase (*p* < 0.05). Furthermore, the yield of breast meat in the T7 group was also significantly enhanced (*p* < 0.05). However, there were no significant differences in semi-eviscerated carcass yield and thigh yield among the groups (*P* > 0.05) (Figures 2B–F).

### 3.3 Effects of FAP on serum antioxidant capacity of chickens

As shown in Figure 3, the experimental mechanism diagram illustrates the process of serum biochemical assays (Figure 3A). Compared to the T1 group, the activities of CAT in the T5, T6, and T7 groups were significantly elevated (*p* < 0.05). Additionally, the activities of GSH-Px and T-AOC levels in the T6 and T7 groups also significantly increased (*p* < 0.05). Compared with the T1 group, the MDA levels in all groups were significantly reduced (*p* < 0.05) (Figures 3B–F).

### 3.4 Effects of FAP on intestinal function in chickens

**Figure 4:**
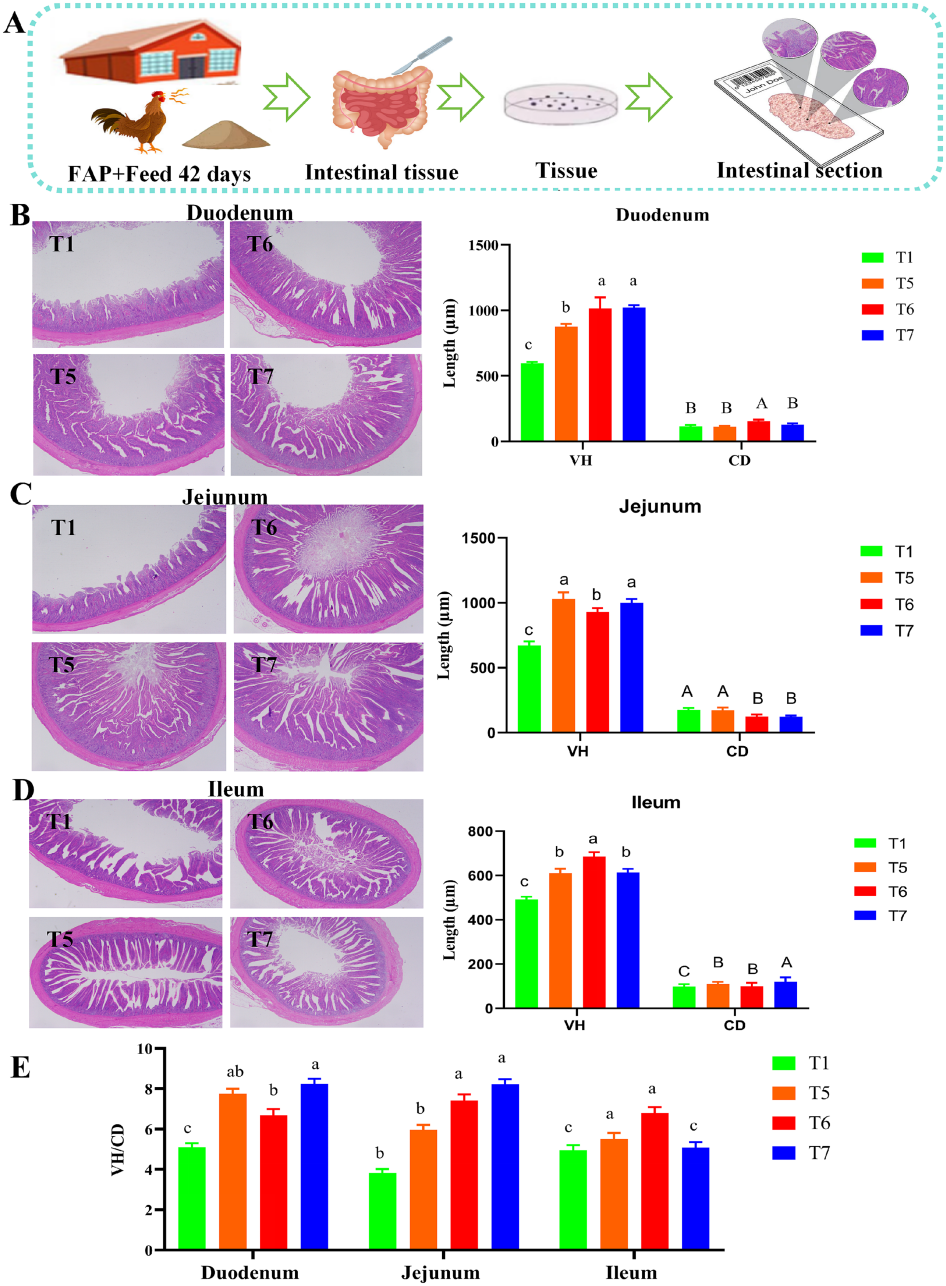
Intestinal morphology of chickens. **(A)** Mechanism diagram of section staining assessment. **(B)** Duodenum structural change of tissue sections. **(C)** Jejunum structural change of tissue sections. **(D)** Ileum structural change of tissue sections. **(E)** VH/CD ratio. Different lowercase letters indicate significant differences (*p* < 0.05, Duncan multiple comparisons).

Figure 4 clearly demonstrates that the supplementation of FAP improved the intestinal villus morphology of broiler chickens. A summary of the effects of FAP on intestinal morphology is presented in the experimental mechanism diagram (Figure 4A). Compared to the T1 group, the VH and VH/CD ratio in the duodenum of the T5, T6, and T7 groups were significantly increased (*p* < 0.05). In the jejunum, VH was significantly increased in the T5, T6, and T7 groups, while CD in the T6 and T7 groups was significantly decreased compared to the T1 group, resulting in an increased VH/CD ratio (*p* < 0.05). Furthermore, in the ileum, both VH and CD were significantly improved in the T5, T6, and T7 groups, with the VH/CD ratio in the T5 and T6 groups also significantly increased (*p* < 0.05) (Figures 4B–D).

**Figure 5:**
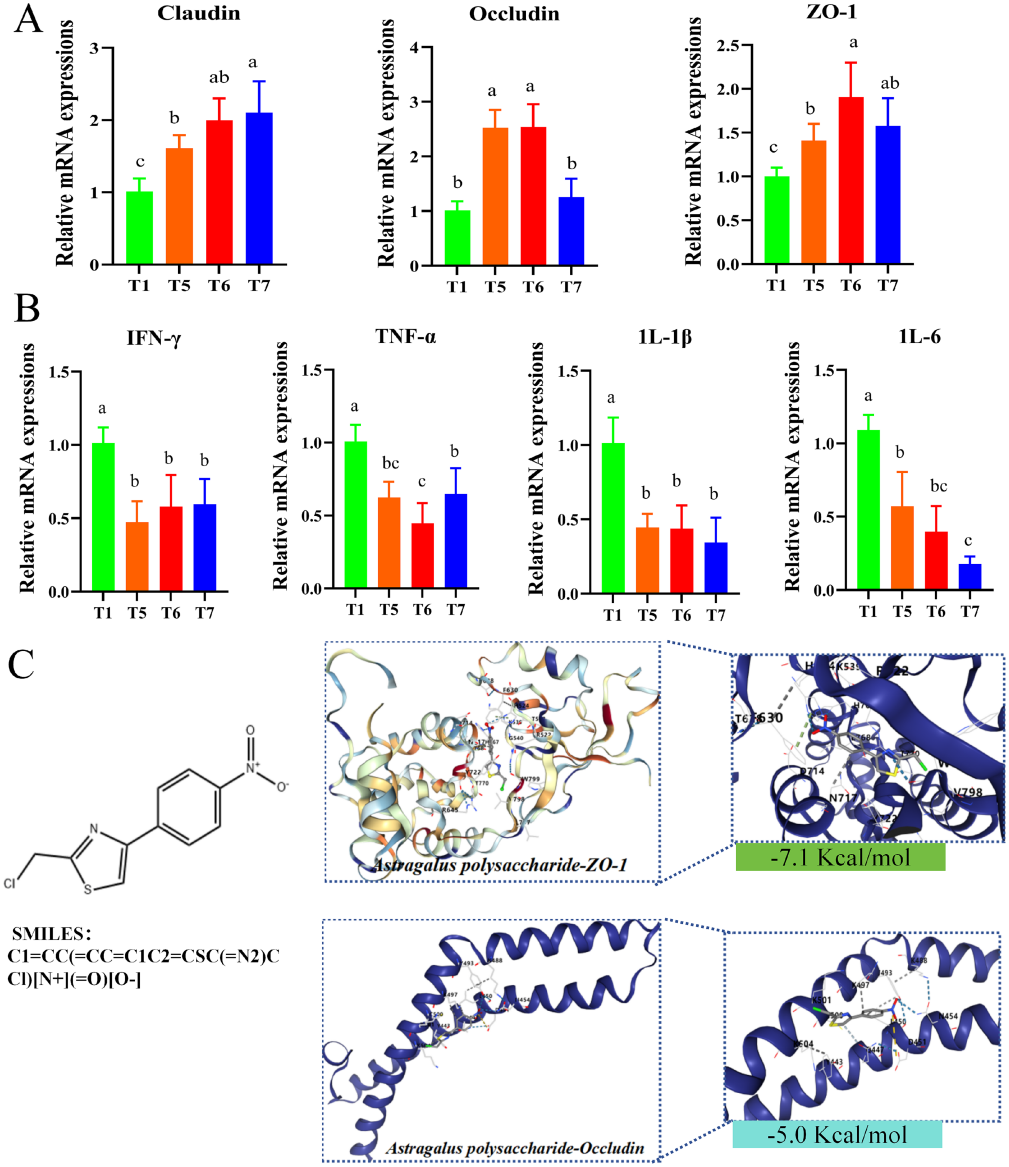
Gene expression in chicken intestines. **(A)** Relative expression levels of *Claudin, Occludin* and *ZO-1* genes. **(B)** Relative expression levels of *IL-1β*, *IL-6*, *TNF-α* and *IFN-γ* genes. **(C)** Molecular docking results of core targets and core components. Different lowercase letters indicate significant differences (*p* < 0.05, Duncan multiple comparisons).

As illustrated in Figure 5, the relative mRNA expression levels of *ZO-1* and *Claudin* were significantly up-regulated in T5, T6 and T7 groups compared with T1 group (Figure 5A). In addition, the relative mRNA expression levels of *Occludin* were significantly increased in T5 and T6 groups (*p* < 0.05) (Figure 5A). On the contrary, the relative mRNA expression levels of *IL-1β, IL-6, TNF-α*, and *IFN-γ* were significantly down-regulated (*p* < 0.05) in T5, T6 and T7 groups compared with T1 group (Figure 5B). Molecular docking experiments were then performed, and the three-dimensional structures of *Occludin* protein and *ZO-1* protein are shown in Figure 5C, indicated by blue bands. The enlarged area demonstrates the binding site of APS with it. The prediction using Swiss Target Prediction revealed that APS can bind well with *Occludin* and *ZO-1*. The relationship between APS and *ZO-1* and *Occludin* was verified practically. The binding energy of APS to *ZO-1* was −7.1 Kcal/mol. Meanwhile, the binding energy of APS and *Occludin* was −5.0 Kcal/mol (Figure 5C). In addition, it can be seen that APS (represented as a ball-and-stick model) forms interactions with specific amino acid residues of *Occludin* proteins (e.g., K504, K501, Y493, D488, R497, R500, R454, K443) (Figure 5C), while interacting with specific amino acid residues of the *ZO-1* proteins (e.g., T630, R645, F630 R624, Y535, R522, G540, N799, D714, N717, V798) to form interactions (Figure 5C). The intermolecular dashed lines represent hydrogen bonding or hydrophobic interactions. These results suggest that these residues may be critical sites for APS to exert its drug effects. It is also an important binding site for improving the function of *Occludin* and *ZO-1* proteins.

### 3.5 The effect of FAP on intestinal microbiota in chickens

**Figure 6:**
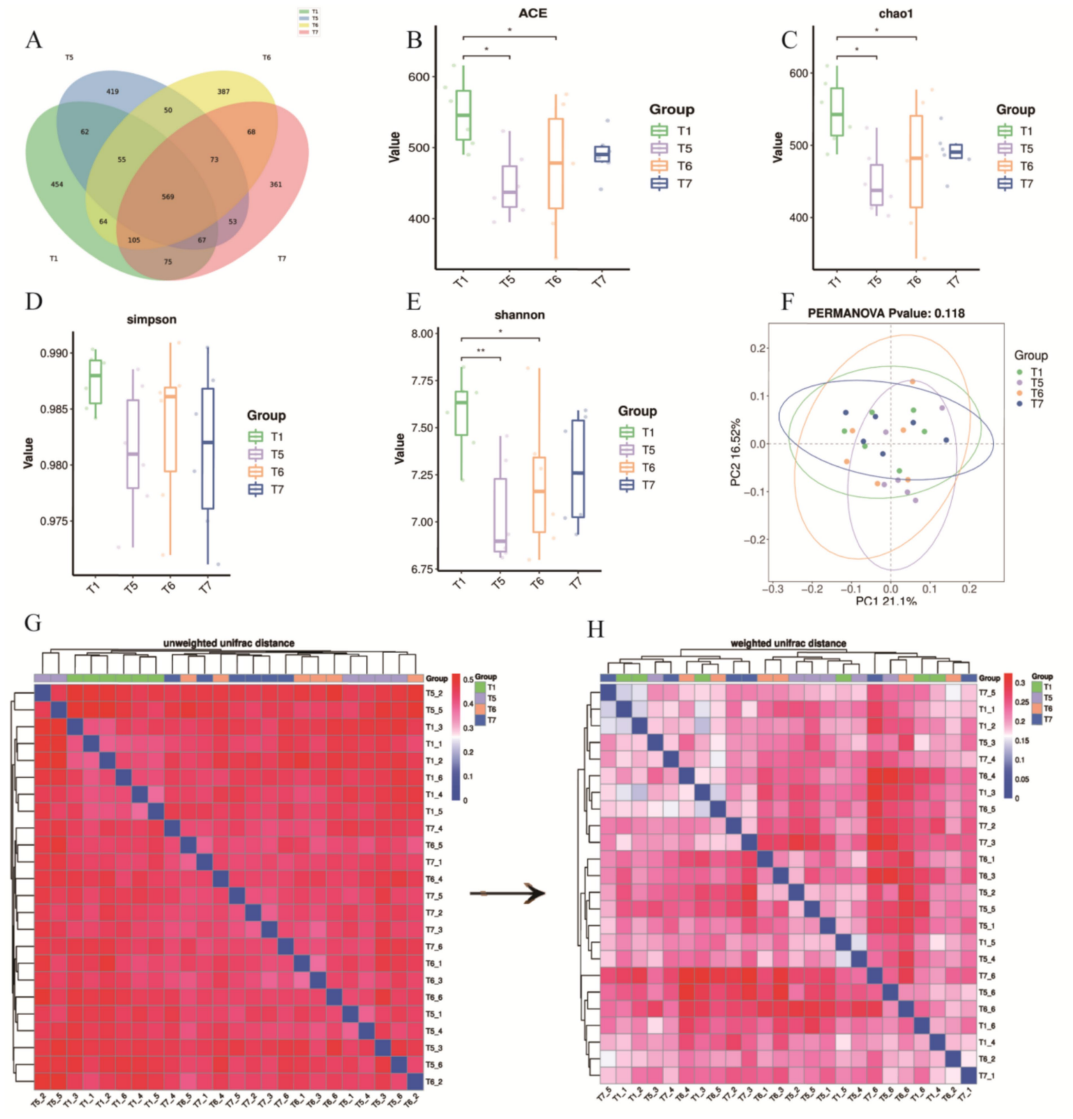
Effects of BECF on the cecal microbiota of broiler chickens/Weighted UniFrac. **(A)** Venn diagram. **(B)** ACE index**. (C)** Chao 1 index. **(D)** Simpson index. **(E)** Shannon index. **(F)** PERMANOVA *p*-value: 0.118. **(G)** Unweighted UniFrac distance. **(H)** Weighted UniFrac distance. Differences between groups are statistically significant, indicated by (*p* < 0.05) and (*p* < 0.01).

This study employed 16S rDNA sequencing to analyze the cecal contents of each group. A total of 2,862 amplicon sequence variants (ASVs) were detected across the four groups, with 569 ASVs found to be shared among all groups. The unique ASVs for the T1, T5, T6, and T7 groups were 454, 419, 387, and 361, respectively (Figure 6A). Bacterial diversity within the cecal microbiota was assessed using Chao1, ACE, Shannon and Simpson indices. Compared to the T1 group, the ACE, Chao1, and Shannon indices of the cecal microbiota in the T5 and T6 groups were significantly lower (*p* < 0.05) (Figures 6B–E). In contrast, there was no statistically significant difference in the Simpson index between groups (*P >* 0.05) (Figure 6D). Beta diversity analysis was conducted using principal coordinates analysis (PCoA) based on weighted UniFrac distance to compare the overall microbial profiles among the groups. The PCoA results indicated that the differences between the two principal coordinates accounted for 37.62%, with PC1 and PC2 explaining 16.52 and 21.1% of the variance in the original dataset, respectively (*p* < 0.05) (Figure 6F).

To further assess differences in microbial community structures, the unweighted UniFrac distance analysis revealed distinct clustering in the unweighted heatmap (Figure 6G). When comparing this with the weighted UniFrac distance, we observed that samples T5, T6, and T7 tended to cluster together, indicating consistency in the treatment effects (Figures 6G,H). Additionally, it was established that abundance information plays a crucial role in evaluating differences among samples.

### 3.6 Effects of FAP on the structure of chicken intestinal microbiota

**Figure 7:**
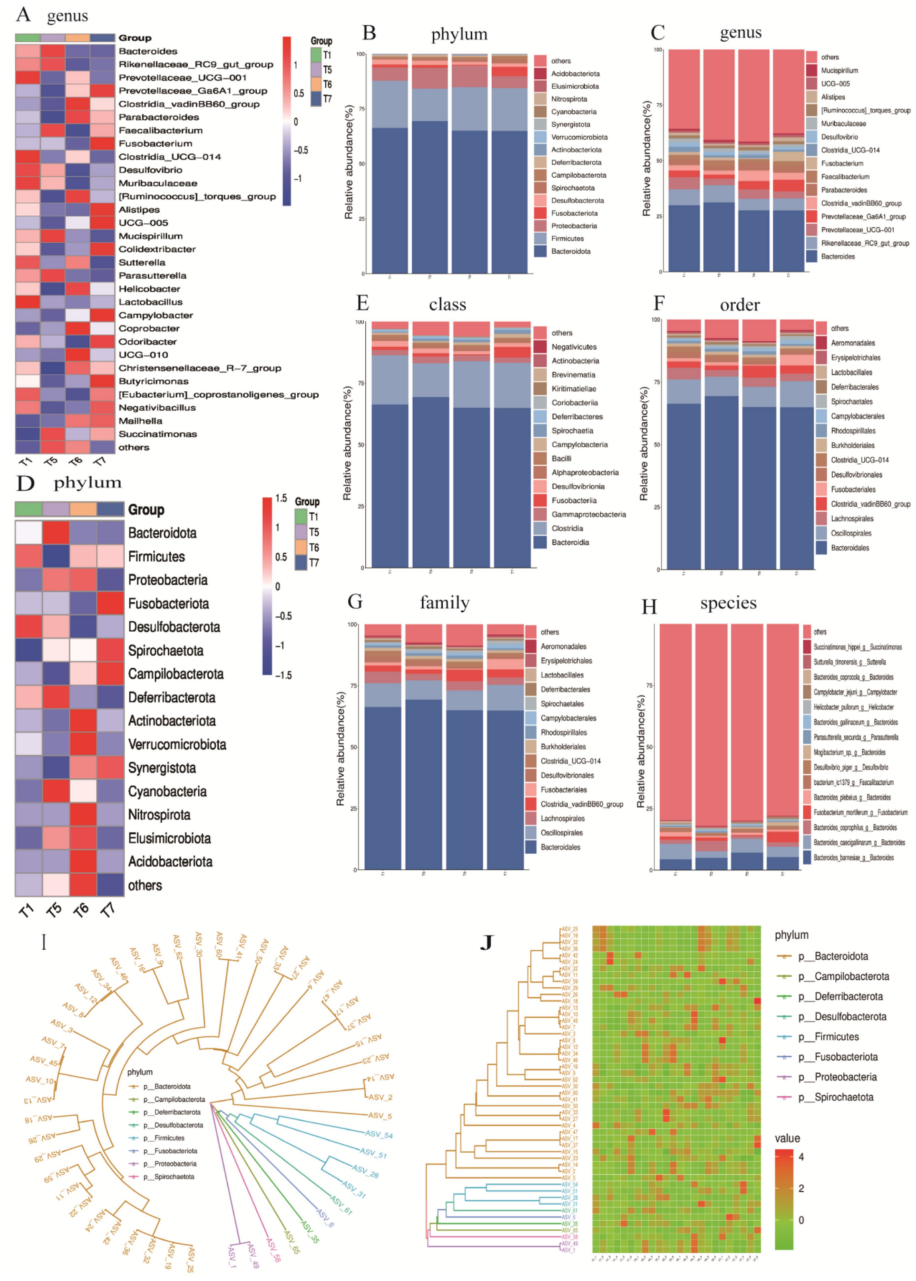
Relative abundance expression at various taxonomic levels. **(A)** Clustering heatmap of the top 30 genera. **(B)** Abundance chart of the top 15 phyla. **(C)** Abundance chart of the top 15 genera. **(D)** Clustering heatmap of the top 15 phyla. **(E)** Abundance chart of the top 15 classes. **(F)** Abundance chart of the top 15 orders. **(G)** Abundance chart of the top 15 families. **(H)** Abundance chart of the top 15 species. **(I)** Phylogenetic tree of the top 50 ASVs. **(J)** Heatmap of the top 50 ASVs.

**Figure 8:**
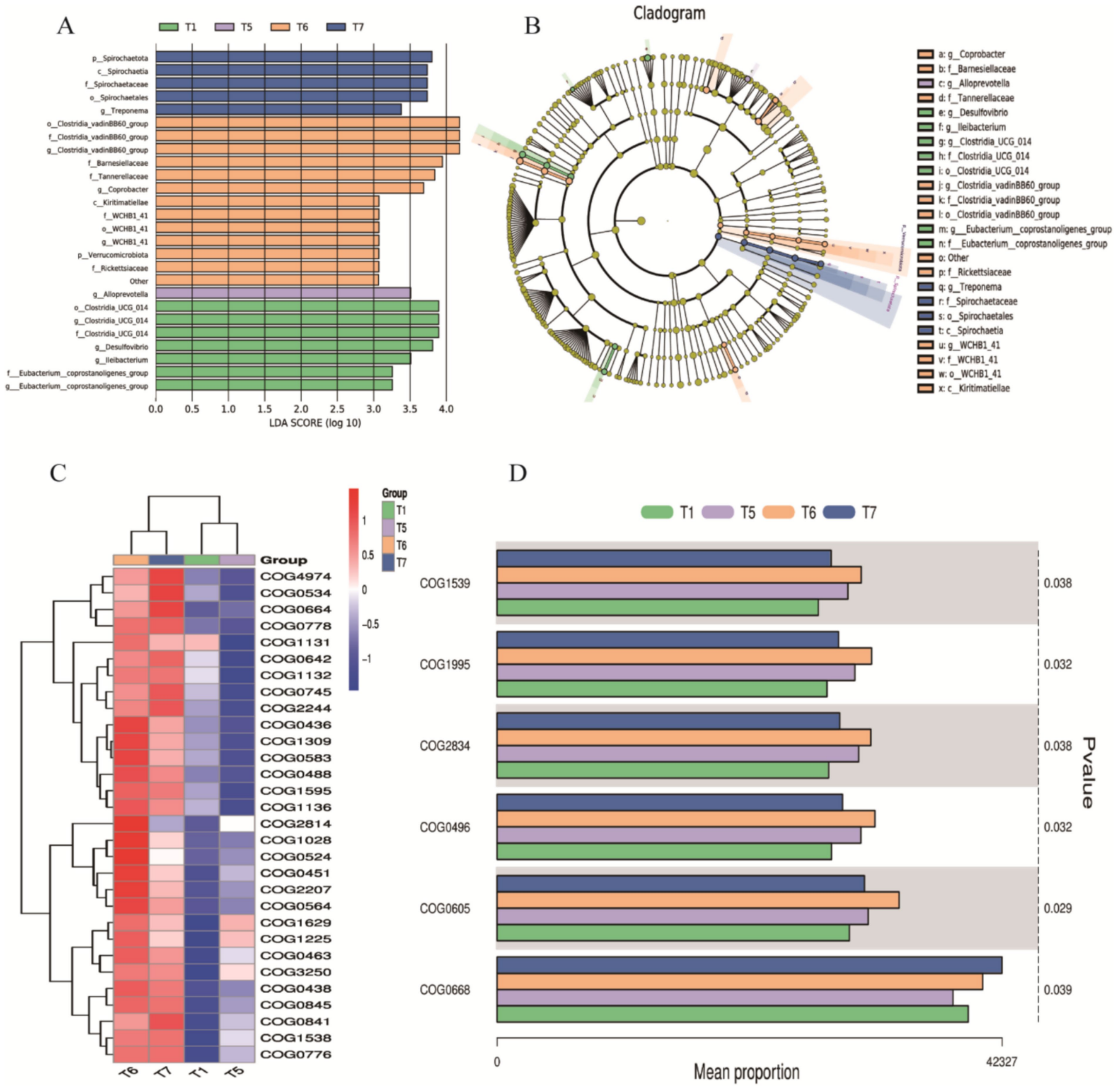
LEfSe/COG Analysis. **(A)** Bar chart of LDA value distribution. **(B)** LEfSe phylogenetic tree: Represents the richness of gut microbiota in chickens from the control group and different proportions of herbal diet groups. Different colors indicate enrichment in different groups, with yellow nodes representing no significant differences; each ring corresponds to a taxonomic level. **(C)** Top 30 COG categories. **(D)** COG abundance analysis based on the Kruskal-Wallis tes.

To assess the impact of FAP on the cecal microbiota, we analyzed the classification and composition of the cecal microbiota at the phylum and genus levels (Figures 7A–D). At the phylum level, the composition of the top 15 phyla was analyzed (Figure 8B), and a clustering heatmap of these phyla was generated for multi-factor analysis. The results indicated that Bacteroidota and Firmicutes were the dominant phyla in the cecum of broiler chickens, accounting for over 84% of the total microbial community. Compared to the T1 group, the abundance of Verrucomicrobiota significantly increased in the T6 group (0.10% vs. 0.27%, *p* < 0.05). Cyanobacteria, Nitrospirota, Elusimicrobiota, and Acidobacteriota were unique to the T6 group, while Cyanobacteria and Elusimicrobiota were unique to the T5 group.

At the genus level, the top 15 most abundant genera were identified (Figure 7C), with dominant genera including *Bacteroide*s, *Rikenellaceae_RC9 gut group, Prevotellaceae_UCG-001, Prevotellaceae_Ga6A1 group, Clostridium vadinBB60 group,* and *Parabacteroides*. The abundance of *Clostridium vadinBB60 group* significantly increased compared to the T1 group, while *Desulfovibrio* decreased significantly (*p < 0.05*). Additionally, the abundance of *Rikenellaceae_RC9 gut group* decreased in the T6 and T7 groups compared to the T1 group, while the abundance of *Prevotellaceae_Ga6A1 group* and *Parabacteroides* increased (*p < 0.05*). All groups showed a decline in the abundance of *Prevotellaceae_UCG-001* compared to the T1 group (*p* < 0.05). A clustering heatmap for the top 30 genera further confirmed that the prominent dominant genera included *Bacteroides*, *Rikenellaceae_RC9 gut group*, *Prevotellaceae_UCG-001*, and *Prevotellaceae_Ga6A1 group*.

At the class level, the dominant microbial communities were primarily concentrated in *Bacteroidia*, *Clostridia* and *Gammaproteobacteria-γ* (Figure 7E). At the order level, the dominant microbial communities were primarily concentrated in *Bacteroidales*, *Oscillospirales*, *Clostridia_vadinBBB60_group*, and *Erysipelotrichales* (Figure 8F). At the family level, the dominant microbial communities were mainly focused on *Bacteroidaceae*, *Prevotellaceae*, *Ruminococcaceae*, and *Tannerellaceae* (Figure 7G). Finally, at the species level, the dominant microbial communities were concentrated in *Bacteroides barnesiae*, *Bacteroides* caecigallinarum, and *Bacteroides coprophilus* (Figure 7H).

To further explore the evolutionary relationships between the classification units (ASVs) and the impact of FAP on gut health and microbial diversity in chickens, we hypothesized that the addition of FAP influenced the structure and composition of chicken gut microbiota at the phylum level. For instance, higher abundances of *Bacteroidota*, *Campilobacterota*, *Deferribacterota*, *Desulfobacterota*, *Firmicutes*, *Fusobacteriota*, *Proteobacteria*, and *Spirochaetota* were observed in the experimental group samples, which may contribute to improving gut health and digestive efficiency in chickens (Figures 7I,J).

To explore the improvement of FAP on specific flora, we performed LDA and LEfSe to visualize the changes in the microbial community structure across different treatment groups. A dendrogram was used to represent the structural and dominant bacteria of the control and experimental groups. The LEfSe analysis (LDA > 3.5, *p* < 0.05) revealed significant differences in microbial taxa among the four groups (Figures 8A,B). The identified biomarkers may hold potential value. In the T1 group, four potential biomarkers were identified: at the phylum level, *Clostridia_UCG_014*; at the family level, *Clostridia_UCG_014*; at the genus level, *Clostridia_UCG_014* and *Desulfovibrio*. In the T6 group, six potential biomarkers were identified: at the order level, *Clostridia_vadinBB60_group*; at the family level, *Clostridia_vadinBB60_group*, *Bacteroidaceae*; at the genus level, *Clostridia_vadinBB60_group* and *Comamonas*. In the T7 group, four potential biomarkers were found: at the phylum level, *Spirochaetia*; and at the class, order, and family levels, all were *Spirochaetia*.

We further conducted COG analysis, with the top 30 COG categories displayed in Figure 8C. The results revealed that certain COG categories had higher abundance in specific groups, particularly *COG01225, COG00841, COG01998, COG00483*, and *COG00304*, which correspond to proteins involved in viral defense or immune responses, proteins participating in inflammatory responses or immune reactions, enzymes involved in redox reactions, key proteins responsible for intracellular signaling, and enzymes or cofactors involved in cellular metabolism. These were significantly expressed in T5, T6, and T7 (Figure 8C). Based on the Kruskal-Wallis test for COG abundance analysis, we identified *COG1539, COG1995, COG2834, COG0496, COG0605*, and *COG0668* related to metabolic enzymes or proteins, functions related to cell membrane and cell wall synthesis or degradation, proteins related to RNA or DNA processing or modification, processes related to cell proliferation or division, and functions involved in protein degradation (Figure 8D). Compared to the T1 control group, experimental groups showed upregulation, particularly in T6 and T7, where significant statistical differences were observed in the abundance of COG categories. These analytical results indicate that the addition of FAP significantly impacts the composition and function of the gut microbiota in chickens, which may have positive effects on gut health and overall welfare.

### 3.7 Effects mechanism of FAP on chicken health

**Figure 9:**
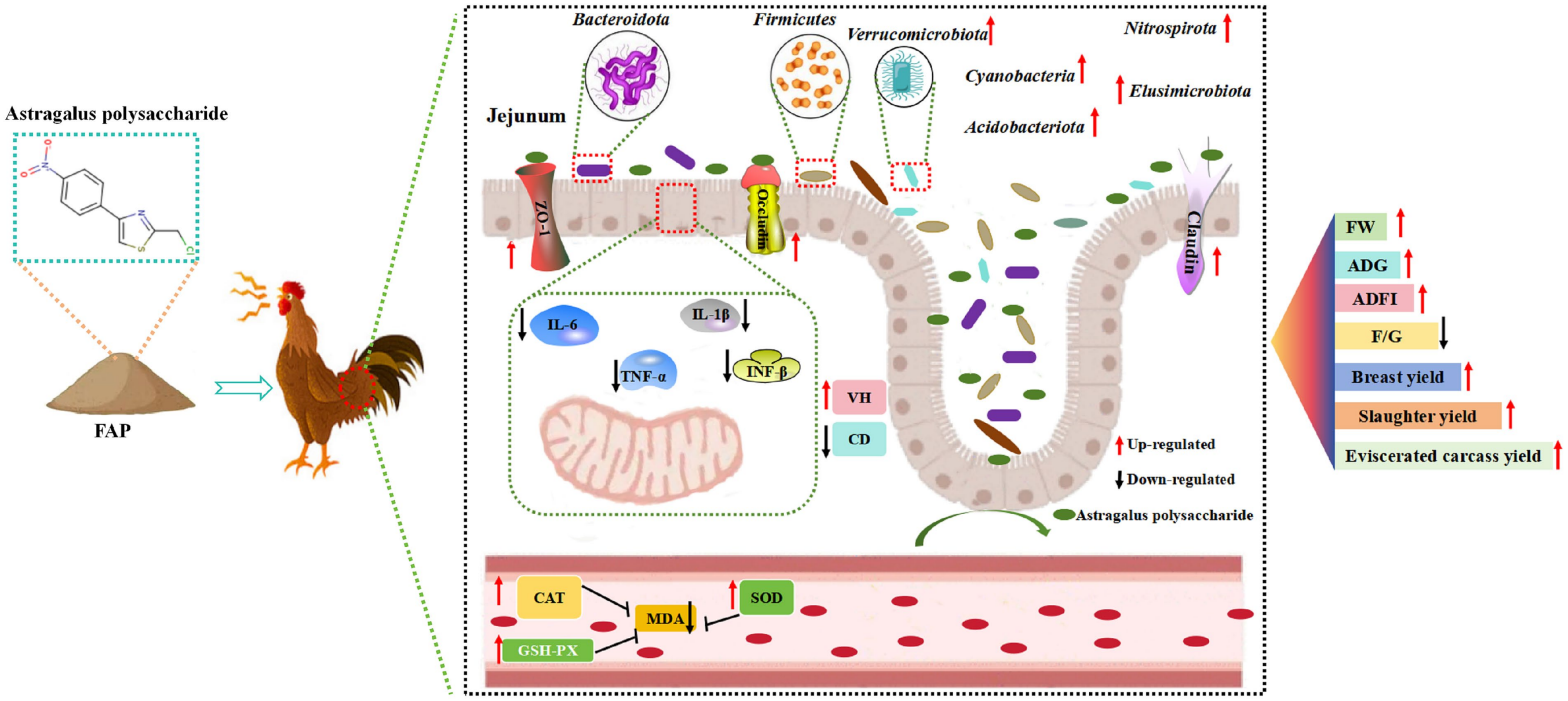
Mechanisms of influence of experimental treatments.

In this experiment, FAP was used as a feed additive to explore its mechanism of action on chicken health (Figure 9). It was found that FAP increased the abundance of major bacterial groups such as *Bacteroidetes, Firmicutes* and *Ferri microbiota* by changing the intestinal microbial community in jejunum, thereby improving the damage of intestinal barrier function caused by microorganisms. At the same time, FAP also enhanced the strong intestinal barrier function of jejunum, further reduced the expression of intestinal mucosal cell inflammatory factors *IL-6, IL-1β, TNF-α* and *IFN-γ* induced by intestinal microorganisms, and alleviated the inflammatory damage of intestinal mucosal cells caused by intestinal microorganisms. In addition, improved intestinal barrier function also reduces the entry of bacterial metabolites into the blood and tissues throughout the body, causing oxidative stress in cells. At the same time, the enhancement of intestinal barrier function also improved the antioxidant capacity of serum, increased the activity of CAT and SOD, and decreased the content of MDA. Overall, FAP improves growth performance and health in chickens through the microbial-gut barrier pathway.

## 4 Discussion

Growth performance index is one of the criteria to evaluate the growth robustness of broilers, and it is also an important index to evaluate the nutritional value and application value of fermented polysaccharide. Slaughter performance accurately reflects the growth condition of animals, with related indicators such as slaughter rate and carcass weight directly indicating the meat production capacity and the economic benefits brought by the animals (22). Previous studies have found that APS can significantly increase ADG in chickens (23). Plant-derived compounds, such as essential oils or polysaccharides, can significantly affect growth performance and immune responses in livestock (24). In addition, Chuanxiong rhizoma is used in the treatment of thromboembolic diseases (25). Adding APS to the diet can significantly reduce F/G (26). Similarly, in this study, it was found that ADG increased and F/G decreased in chickens fed FAP. In addition, this study also found that the addition of FAP can significantly increase slaughter yield, exsanguinated carcass yield and breast yield. Previous studies have also found that APS can improve chicken carcass quality (27, 28). This may be because the FAP improves the intestinal flora structure and increases the abundance of probiotics, thereby improving carcass quality. Although the effect of Cyan-shank Partridge chickens cannot fully represent all other broiler breeds, its effect as one of the broiler breeds can be used as a reference for other breeds to a certain extent. In particular, the impact of the Cyan-shank Partridge chickens is used as a model to provide basic data and theoretical support for further research in other breeds. For example, in order to solve the problem of poor gel performance in the marine organism giant squid (29).

In organisms, free radicals and the antioxidant system exist in a balanced state, which is essential for maintaining animal health. Antioxidant enzymes play a key role in combating oxidative stress, primarily including CAT, SOD, and GSH-Px. These enzymes can neutralize metal ions, free radicals, and reactive oxygen species, preventing peroxide formation and mitigating lipid peroxidation, thereby achieving an antioxidant effect (30, 31). Additionally, MDA serves as an important indicator of oxidative stress, with its concentration reflecting the extent of oxidative damage experienced by the body (32). Studies have shown that APS can increase the activity of SOD and GSH-Px in serum, and reduce the concentration of MDA, so as to improve the antioxidant capacity of the body (33). Similarly, our study found that the addition of fermented Astragalus polysaccharide can increase the activities of CAT and GSH-Px and T-AOC levels in serum of broilers, while decrease the content of MDA in serum, and ultimately enhance the antioxidant capacity of broilers. The results showed that the fermentation of APS had high growth performance.

The small intestine of broilers is a crucial site for nutrient absorption and plays a significant role in the digestion, absorption, and transport of nutrients. The VH and CD of the small intestine are important indicators for assessing its digestive and absorptive capacity. When the intestinal villi lengthen, the contact area between the intestinal mucosa and the intestinal chyme increases, thereby enhancing the small intestine’s ability to digest and absorb nutrients (34). The crypt depth reflects the rate of cell proliferation, a shallower crypt depth indicates a higher maturation rate of intestinal epithelial cells and an enhanced ability to absorb nutrients (35). Therefore, the ratio VH/CD can comprehensively reflect the digestive and absorptive functions of the small intestine. An increased ratio indicates enhanced digestive and absorptive functions, whereas a decreased ratio affects the small intestine’s ability to digest and absorb nutrients. Related studies have found that dietary supplementation of APS can increase intestinal VH and VH/CD of poultry, and improve intestinal structure and function (36). Similar to the results of previous studies, the results of this study showed that feeding FAP could increase intestinal VH and CD and improve intestinal morphology of broilers.

Intestinal epithelial cells act as a crucial physical barrier against external environmental stimuli, and tight junction proteins are the primary structural proteins that constitute the intestinal epithelial barrier function (37). These proteins are mainly composed of three types of transmembrane proteins: *ZO-1*, *Claudin*, and *Occludin*. *ZO-1* is a group of scaffold proteins that form part of the tight junction proteins. *Claudin* and *Occludin* are essential components in regulating intestinal epithelial barrier function, sealing intercellular gaps, determining the selective permeability of intestinal epithelial cells, and thereby playing a role in regulating intestinal barrier function and improving intestinal health. Damage to intestinal tight junctions leads to a compromised intestinal barrier structure and increased intestinal permeability (38). Inflammatory responses can increase the basal metabolic rate of animals, elevate protein degradation rates, reduce synthesis rates, and consequently impact animal growth. *IL-6* and *TNF-α* are major pro-inflammatory factors in the body. *IL-1β*, a significant member of the *IL-1* family, plays an important role in inflammation-related diseases. *IL-1β* has strong pro-inflammatory activity and can induce various pro-inflammatory mediators, such as cytokines and chemokines, ultimately leading to an amplified inflammatory response (39). Local activation of *IL-1β* is central to pro-inflammatory responses and can activate secondary inflammatory mediators, including *IL-6*. Similar to I*L-1β*, *TNF-α* is a multifunctional pro-inflammatory cytokine belonging to the *TNF* family. *TNF-α* plays diverse roles in regulating development and immunity, including inflammation, differentiation, lipid metabolism, and apoptosis, and is associated with various diseases (40). *IFN-γ*, a soluble dimeric cytokine, is the sole member of type II interferon, and its overactivation can cause tissue damage, necrosis, and inflammation. The results of this study showed that feeding FAP could up-regulate the relative expression level of tight junction protein-related gene mRNA in jejunal mucosa and down-regulate the relative expression level of jejunal pro-inflammatory factor mRNA in broilers, thereby improving intestinal barrier function. Similar to previous studies on APS (41). It has been shown that FAP can improve intestinal barrier function, enhance intestinal development and maintain intestinal health. In addition, the molecular docking test of *ZO-1* and *Claudin* protein showed that FAP had strong binding properties to *ZO-1* and *Claudin* protein. At the same time, FAP components can combine with *D714* and *N717* amino acid residues in *ZO-1* protein, and FAP components can also bind *K497, Q447* and *L450* residues of Occludin, which may be the key sites for APS to exert its drug function. Therefore, it is speculated that the binding effect of FAP with these residues (*K497, Q447, L450, D714,* and *N717*) is the key to its intestinal barrier function.

The intestinal microbiota of poultry represents a vast microbial community that significantly influences their health and productivity (42). This microbiota plays a critical role in promoting food digestion, maintaining immune balance, and resisting pathogenic invasion. Additionally, it regulates the physiological and biochemical responses in poultry, thereby accelerating the digestion and absorption of nutrients while enhancing immunity (43). Maintaining the equilibrium of intestinal microbiota is crucial for ensuring microbial diversity and stability. However, the composition and ecological succession of the intestinal microbiota in poultry are affected by various factors, including diet composition, environmental conditions, and genetic background (44, 45). Among these factors, diet composition is a key determinant influencing the configuration of the intestinal microbiota (46). After feeding broilers with FAP, the cecal microbiota was analyzed using 16S rDNA sequencing, revealing that the use of FAP significantly reduced the ACE, Chao1, and Shannon diversity indices of the cecal microbiota. This decrease in microbial diversity may be attributed to the colonization of certain dominant bacterial populations, which can hinder the establishment of other bacteria, including harmful species (47). Previous studies have also demonstrated significant reductions in the ACE, Chao1, and Shannon indices of the intestinal microbiota in broilers following the use of fermented feeds (48). At the phylum level, *Bacteroidota* and *Firmicutes* were identified as the predominant phyla, dominating the intestinal microbiota. This suggests that the incorporation of FAP may lead to an increase in their abundance, facilitating improvements in intestinal health and digestive efficiency (49). Notably, the abundance of *Verrucomicrobiota* was significantly elevated in the T6 group compared to the T1 group, and the presence of *Cyanobacteria*, *Nitrospirota*, *Elusimicrobiota*, and *Acidobacteriota* was unique to the T6 group. Some of these bacteria confer benefits to the host, promoting the growth and maintenance of the intestinal mucosal layer, enhancing intestinal barrier function, reducing the growth of harmful bacteria, and lowering intestinal toxin levels (50). These functions help protect intestinal health and support the host’s glucose homeostasis, exhibiting anti-inflammatory properties. For instance, bacteria from *Verrucomicrobiota* can degrade polysaccharides through specific enzymes such as fucosidases and sulfatases, thereby improving the host’s nutrient absorption capacity (51). Additionally, members of *Elusimicrobiota* can promote intestinal health by producing short-chain fatty acids (SCFAs), which serve as an energy source for the host and possess anti-inflammatory effects. At the class level, *Bacteroidia* and *Clostridia* are two significant classes within the phylum *Firmicutes*. Further classification at the order level revealed an increase in the abundance of *Bacteroidales* and *Oscillospirales* in the poultry intestinal microbiota, aiding in the identification of microorganisms occupying specific ecological niches. Bacteria from these two classes are involved in protein metabolism by hydrolyzing proteins into peptides and amino acids. This process not only facilitates the digestion and absorption of proteins in poultry but also impacts amino acid balance and overall metabolic health (52). Further analysis at the family level indicated an increased abundance of *Bacteroidaceae* and *Prevotellaceae*, which positively influence poultry health and performance through enhanced carbohydrate metabolism, immune modulation, and anti-inflammatory actions (53). For example, bacteria from the *Bacteroidaceae* family can interact with the host’s immune system by producing polysaccharides, subsequently promoting the differentiation of regulatory T cells and enhancing host immune tolerance. This interaction helps maintain intestinal immune balance and reduces inflammatory responses (54). This includes the study by Huang et al. who explored the species-specific effects of microbiota on gut health, which is consistent with the microbial changes observed in this study (55). In contrast, bacteria from the *Prevotellaceae* family can mitigate intestinal inflammation by producing anti-inflammatory metabolic products, with the *Prevotella* genus being particularly effective in degrading complex polysaccharides and promoting energy acquisition (56–58).Overall, the above changes in microbial community abundance reflect functional enhancements in energy metabolism, gut barrier nutrient absorption, and immune regulation, and it is worth noting that the gut flora can communicate bi-directionally with the host’s nervous system via the gut-brain axis. May be associated with systemic benefits of improved gut health (59). For example, the growth of cyanobacteria increases the variety and content of neuroactive substances, further enhancing the signaling of the gut-brain axis.

Moreover, a COG functional classification analysis related to intestinal health revealed higher abundances of COGs associated with metabolic enzymes or proteins, including *COG1539, COG1995, COG2834, COG0496, COG0605,* and *COG0668* within the microbial community. The expression of these COGs in the poultry intestinal microbiota may enhance digestive efficiency and immune status. They are also involved in pathways related to viral defense, immune responses, redox reactions, intracellular signal transduction, and cellular metabolism. For example, *COG1539* pertains to key enzymes or proteins in cellular metabolic processes, while *COG0605* relates to the transport and metabolism of inorganic ions, being associated with SOD, an enzyme that plays a crucial role in mitigating oxidative stress and scavenging free radicals. Additionally, LEfSe analysis indicated that groups T1, T6, and T7 contained 4, 6, and 4 potential biomarkers, respectively. These identified biomarkers may possess significant value, but further research is required to elucidate their role in modulating gastrointestinal health in poultry. IIn conclusion, the analysis of the cecal microbiota suggests that the use of FAP may regulate intestinal health by balancing the intestinal microbiota.At the same time, the potential challenges of promoting the use of FAP currently require the investment of a large amount of money for basic research, clinical trials and other aspects of validation, presenting the difficult nature of increased costs, complex and stringent approval processes with uncertainty of benefits. We hope to further address these challenges in the future.

## 5 Conclusion

This study showed that feeding FAP could improve the growth performance, slaughtering characteristics, immune capacity and antioxidant capacity of broilers by regulating the microbial-intestinal barrier pathway. Then FAP can improve the health condition of chickens.

## Data Availability

The datasets presented in this study can be found in online repositories. The names of the repository/repositories and accession number(s) can be found below: https://www.ncbi.nlm.nih.gov/; PRJNA940200.

## Footnotes

^1^ https://www.rcsb.org/
